# The Intertwined Relationship Between Malnutrition and Poverty

**DOI:** 10.3389/fpubh.2020.00453

**Published:** 2020-08-28

**Authors:** Faareha Siddiqui, Rehana A. Salam, Zohra S. Lassi, Jai K. Das

**Affiliations:** ^1^Division of Women and Child Health, Aga Khan University, Karachi, Pakistan; ^2^Robinson Research Institute, Adelaide Medical School, University of Adelaide, Adelaide, SA, Australia

**Keywords:** malnutrition, poverty, undernutrition, obesity, food insecurity

## Abstract

Despite social and economic development, the burden of malnutrition across the globe remains unacceptably high. A vital relationship exists between nutritional status, human capital, and economic standing. Malnutrition adversely affects the physiological and mental capacity of individuals; which in turn hampers productivity levels, making them and their respective countries more susceptible to poverty. A two-way link exists between malnutrition and poverty, creating a vicious cycle with each fueling the other. Malnutrition produces conditions of poverty by reducing the economic potential of the population and likewise, poverty reinforces malnutrition by increasing the risk of food insecurity. The aim of the paper is to describe the interconnection between malnutrition and poverty, and to highlight how both serve as the cause and consequence of each other. The paper also discusses ways to move ahead to tackle these issues in a parallel manner rather than in separate silos.

## Introduction

Malnutrition relates to a deficiency, excess, or imbalance of energy and other macro and micro-nutrients. It comprises of varying degrees of under- or over- nutrition, which leads to changes in body composition, body function, and clinical outcomes. In other words, malnutrition is an all-inclusive term that represents all manifestations of poor nutrition and ranges from extreme hunger and undernutrition to obesity (1, 2). Despite social and economic development, the burden of malnutrition across the globe remains unacceptably high (2), recent data suggests that ~800 million people are undernourished, out of which 780 million reside in low-to-middle income countries, especially in Sub-Saharan Africa and South Asia (2). In 2015, inadequate food intake and poor dietary quality were responsible directly or indirectly for causing ill-health with six of the top 11 global risk factors being associated with dietary imbalances (2) and in 2017, 11 million deaths and 255 million disability-adjusted life years (DALYs) were attributable to dietary risk factors (3). Children under the age of 5 years are highly vulnerable to malnutrition with estimates suggesting that in 2019, globally 144 million children under the age of five were stunted (short for his/her age), 47 million wasted (thin for his/her height) and 38 million overweight (abnormal or excess bodyweight) (4). In adults, obesity is becoming more prevalent worldwide with ~38.9% of the adult population being either overweight or obese (5). Paradoxically, even though women have a higher prevalence (15.1%) of obesity than men (11%) (5); millions of women around the world are still underweight and one-third of women of reproductive age are estimated to have anemia (5).

Malnutrition has long been linked to poverty as higher rates of malnutrition are found in areas with chronic poverty (6). The impact of poverty on individuals can be seen through multiple manifestations and includes poor nutritional status, food insecurity, vulnerability to disease, reduced productivity levels, and compromised physical and intellectual development. Additionally, people living in poverty are unable to access necessities including nutritious food, hygienic environment, appropriate shelter, and adequate health care (7). Therefore, it would not be incorrect to suggest that even though malnutrition is a global phenomenon, those living in poverty face a higher burden. The question that now arises is whether malnutrition is a cause or consequence of poverty. The relationship between the nutritional status and economic standing has been further explored through the course of this paper.

## Poverty

The World Bank has set the International Poverty Line at $1.90 per person per day using 2011 Purchasing Power Parity (PPP) conversion factors (8). Therefore, households with a per capita income or expenditure less than the standard poverty line are defined as being poor (9). This makes income level the prime indicator for poverty, however with the passage of time, the need for re-conceptualizing poverty is becoming more evident as poverty is complex and multifaceted. Therefore, the conceptualization of poverty should not be limited to average income and wealth only but should encompass various other deprivations that are often experienced by people living in poverty. The global Multidimensional Poverty Index (MPI) is an international measure of acute poverty covering over 100 developing countries; created by the Oxford Poverty and Human Development Initiative (OPHI) and the United Nations Development Programme (UNDP) in 2010 (10). The global MPI steps away from the traditional view of poverty being solely limited to average income and wealth; to a more holistic view that highlights the need for using multiple indicators to account for various issues faced by people as a consequence of poverty (10). Through this index, poverty is portrayed to be a deprivation of basic amenities that restricts individuals from leading a good and healthy life (11) and takes into account the systemic disparities within a country and stretches the boundaries of poverty beyond the shortage of material assets to a concept that encompasses multiple deprivations, including but not limited to: assets, living standards, education, sanitation and hygiene, health and nutrition (10).

Since the 1990s, it is estimated that the proportion of the world's population living in extreme poverty has declined by more than a half (8). In 2015, 10% of the world's population lived under the poverty line; compared to nearly 36% in 1990 (8). Unfortunately, despite the overall decline in global poverty, progress has been uneven and disproportionate with the majority of the world's poor residing predominantly in Sub-Saharan Africa and South Asia (8). In 2015, 736 million people lived in extreme conditions of poverty with half of them i.e., 368 million residing only in five countries of India, Nigeria, Democratic Republic of Congo, Ethiopia, and Bangladesh (8). This illustrates that certain countries especially those afflicted by conflict, poor governance, and natural disasters continue to experience a skewed burden of poverty.

To analyze the vital linkages between poverty and malnutrition; it is important to highlight the growing evidence that health outcomes including malnutrition are driven by social determinants of health i.e., the conditions and circumstances in which people live, learn, work, and even play have a significant impact on their health (12). This interconnection between people's conditions and circumstances and their health can be displayed using the concept of poverty and food insecurity. The term “food insecurity” refers to a situation in which people do not have adequate physical, social or economic access to sufficient and nutritious food (13). Broadly, food insecurity is assessed using four dimensions i.e., food availability, access to food, stability of supply and safe, and healthy food utilization (14). Food insecurity may occur at various levels including regional, national, household, or individual. Poverty and food insecurity are deeply related, as poverty may adversely affect the social determinants of health and may create unfavorable conditions in which people might experience unreliable food supply (13). Food is a major household expenditure for the poor households (15). Data from African countries indicate that close to half of household income is spent on food: Nigeria (56.4%) (16); Kenya (46.7%), Cameroon (45.6%), Algeria (42.5%) (17). Similarly, within high-income countries, low-income households spend a significant proportion of their income on food: Ireland (14–33%), USA (28.8–42.6%) (17, 18). In comparison, the wealthiest households in the USA spend a much lower 6.5–9.2% of household income on food (17). Despite spending a large proportion of their household income on food, many poor households continue to remain food insecure because of their insufficient, irregular, and fluctuating incomes (2, 13).

## Poverty, Food Insecurity and Double Burden of Malnutrition

A vital relationship exists between malnutrition and poverty. Poverty creates unstable and unfavorable conditions that may contribute to fueling the problem of malnutrition (7). People living in poverty often face financial limitations, which hinders their ability to access safe, sufficient, and nutritious food (7). Food insecurity compromises people's ability to acquire the amount of food needed to fulfill the bodily requirement of calories and without sufficient calorie intake, an individual may not be able to build up energy or strength to carry out everyday life activities and this also hampers the capacity and productivity to earn (19). While people living in poverty may require a greater quantity of food than they cursrently have, it is important to take into consideration that appropriate intake of nutrients and quality of food is equally important (19). Poverty can contribute to worsening malnutrition by compromising the quality of food intake and bolstering hidden hunger which is the deficiency of essential vitamins and minerals. The burden of obesity has extended beyond wealthier, developed nations and has now also become a feature of the developing world (16). Poverty leads to financial constraints that in turn lead to the consumption of cheap, high-energy staple foods, primarily carbohydrates, and fats rather than nutritionally dense food. Through the consumption of carbohydrates and fats, energy levels spike; but nutritional quality becomes compromised. The consequence of this is reduced nutritional quality and nutrient deficiencies. Poverty plays a significant role in regulating access and preference of foods (13, 16), and this is evident in studies that showcase that when people living in poverty get a chance to spend relatively more on food; they often prefer to buy better tasting food, rather than good quality food (19).

The deficiency of micronutrients or “hidden hunger” is an important component of malnutrition (13). Micronutrient deficiencies can exists in all age groups and in any socioeconomic bracket. Iron, folate, vitamin A, iodine, and zinc deficiencies are among the most common and widespread micronutrient deficiencies among women and children in low- and middle- income countries and many of these micronutrient deficiencies co-exist. Assessing the relationship between malnutrition and poverty, requires consideration of micronutrient deficiencies. While macro- and micro- nutrient deficiencies may cause suboptimal mental and physical development, recurrent infections and growth retardation (20, 21); micro-nutrient deficiencies may also result in adverse birth outcomes including low birth weight babies (22, 23). To date, ~20 million babies are born with low birth weight each year and there is growing evidence of the connections between slow growth in height early in life and impaired health and educational and economic performance later in life (5, 24). Low birth weight in babies can contribute to the vicious cycle of malnutrition since maternal nutrition status especially maternal stature has been reported to be inversely associated with offspring mortality, underweight, and stunting in infancy and childhood (22, 23). Moreover, the importance of adequate intake of micronutrients can be noted in children born to mothers with sufficient amounts of iodine during pregnancy (19), as these children tend to complete one-third or one-half a year more schooling than children born to mothers with inadequate amount of iodine during pregnancy (19). It has been suggested that if every mother took iodine capsules during pregnancy then this could improve educational attainment among children in Central and Southern Africa (19).

Briefly put, the double burden of malnutrition and the importance of micro-nutrients should be recognized when analyzing the malnutrition-poverty cycle. There is a growing need to reimagine the concept of malnutrition and development experts and policy makers should make strides to account for the inherent complexities of both concepts in order to develop successful and sustainable nutritional strategies (19).

## Malnutrition: Cause or Consequence of Poverty?

The question that now arises is whether malnutrition is a cause or consequence of poverty and vice versa? To elaborate upon this, it is important to highlight the relationship of human capital with nutrition and poverty.

Human capital is an integral asset of any country and the process of developing human capital begins from infancy and continues throughout the course of an individual's life (25). Nutritional status has a profound impact on human capital. The reasoning is simple, improved nutritional status is vital for escaping poverty, as good health is needed to increase productivity levels, contribute to economic growth, and improve a country's overall welfare (6). Without adequate nutrition, human capital starts to decline. This is because malnutrition negatively impacts physical and mental development, intellectual capacity, productivity, and the economic potential of an individual (25). As a consequence, economic stability is threatened, making a country more vulnerable to poverty. Poverty contributes to the problem of food insecurity which is referred to as a “resourced-constrained” or “poverty related” condition. Although the populations affected by poverty and food insecurity overlap; it is important to note that not all people living in poverty are food insecure and that this problem also exists in people living above the poverty line. Moreover, poverty also contributes in creating conditions of micro-nutrient deficiencies and hidden hunger. These factors exacerbate the issue of malnutrition and makes individuals more vulnerable to other health concerns. Irregular and unstable food supply along with low quality of food due to insufficient or inadequate nutrient intake can compromise immunity and make individuals more susceptible to infections. Additionally, if infected, matters tend to become worse because infections may further reduce nutritional and health status, thereby aggravating malnutrition and reinforcing its cycle with poverty (25, 26).

A vicious cycle exists through which both poverty and malnutrition fuel and reinforce each other (25). Globally, the poorest countries are the countries bearing the highest burden of malnutrition. Nutritional imbalances reduce work capacity and human capital; and this makes countries more susceptible to poverty. Furthermore, malnutrition is also a consequence of poverty, as poverty increases food insecurity and hidden hunger; which contributes to the problem of malnutrition. This makes both these elements a cause and a consequence of each other. Establishing a linear relationship between the two would overlook the complexities and nuances that exist within the framework of this topic.

## What Will be the Next Steps?

In order to progress socially and economically, there is an urgent need to recognize the burden of poverty and malnutrition and to take immediate steps to break the ongoing cycle. To achieve this target, it is important to understand what factors feed and reinforce it.

The cycle of poverty and malnutrition appears to be intergenerational. Evidence suggests that malnourished women are at a higher risk of having malnourished children and this creates an intergenerational effect (6). It is imperative to intervene early in life in order to maximize the effectiveness of interventions and break the cycle. The Lancet Nutrition Series (27) modeled the effect of 10 evidence based nutrition specific interventions on lives saved in the 34 countries that have 90% of the world's children with stunted growth. The series also examined the effect of various delivery platforms and delivery options using community health workers to engage poor populations and promote behavior change, access to and uptake of these interventions. Findings suggest that the current total of deaths in children younger than 5 years can be reduced by 15% if populations can access these 10 evidence-based nutrition interventions at 90% coverage. These nutrition specific interventions included salt iodization, multiple micronutrient supplementation in pregnancy (includes iron-folate), calcium supplementation in pregnancy, energy-protein supplementation in pregnancy, vitamin A supplementation in childhood, zinc supplementation in childhood, breastfeeding promotion, complementary feeding education, complementary food supplementation, and management of severe acute malnutrition in children. The findings also support the use of various community engagement and delivery strategies that can help reach poor segments of the population at greatest risk in order to make a difference (27). In other words, the interventions need to reach the poorest of the poor to break the cycle of malnutrition and poverty and should also incorporate disease and infection prevention as a part of their strategy (25).

Considering the inter-linkages described above between malnutrition and poverty, nutrition specific interventions need to be augmented with nutrition sensitive interventions in order to accelerate the progress of reducing malnutrition. Nutrition sensitive interventions are those that address intermediate and underlying causes of malnutrition and help to improve access to nutritious food, clean water and sanitation, education and employment, and health care etc. Large scale nutrition programs focusing on evidence based nutrition interventions should also target key underlying determinants of nutrition including poverty in order to enhance the coverage and effectiveness of nutrition-specific interventions. These include interventions in the sectors of agriculture, social safety nets, early child development, education, and women's empowerment. Women's empowerment is instrumental in not only improving malnutrition but general well-being (28). Hence, a parallel focus on nutrition sensitive and nutrition specific interventions has the potential to greatly accelerate progress in not only the areas of nutrition but also break the intergenerational cycle of malnutrition and poverty (29). More recently, bio-fortification and agricultural biodiversity are also considered to have the potential to cater to the issues of poverty and malnutrition in a parallel manner (30). In developing countries, bio-fortification could focus on improving quality of coarse cereals, as well as fodders along with community participatory approaches to enhance agricultural biodiversity. This approach not only could contribute to a reduction in malnutrition and poverty, but reduce food insecurity and improve sustainability (31, 32), though further research is needed in the domain (30, 31). Income disparity is also a factor that allows the malnutrition-poverty cycle to persist. In fact, a country may experience economic growth, but still have widespread poverty and high levels of malnutrition. This is because income inequality translates as health inequality; as the income gap grows, so does health disparity (7, 13, 25). Furthermore, gender inequities have also been associated with both poverty and malnutrition as a result of lower opportunities for women in the fields of education and employment. A recent analysis based on data from 49 low- and middle-income countries assessing the relationship between gender equity and malnutrition and health suggests that gender equity in education and employment decreases child malnutrition and is an important determinant in nutrition and access to health care (33). Therefore, any attempt to improve global nutritional status and to achieve the targets set by the “2030 Agenda for Sustainable Development” requires a focus on alleviating poverty and simultaneously focusing on agriculture, social safety nets, early child development, education, and strengthening women's position in society (34–37).

Nutritional interventions should be designed in an all-rounded, holistic manner. It would be fruitful to involve multiple stakeholders including health, education, agriculture, water, sanitation and hygiene, gender and economics. To ensure sustainability, nutritional interventions should be context-specific and should also be cost-effective since these issues concern low and middle income countries.

## Conclusion

Ending poverty in all its forms is the first of the 17 Sustainable Development Goals and ending hunger, reducing food insecurity and improved nutrition and agriculture is the second goal. Furthermore, at least 12 of the 17 goals contain indicators that are highly relevant to nutrition. Poverty and malnutrition are deeply interrelated, with each fuelling the other and hence it is imperative to tackle both issues simultaneously rather than in parallel silos. A two-way link exists, with both elements being the cause and consequence of each other. This vicious cycle remains a prime public health concern and immediate strides need to be made against it. For a sustainable improvement in nutritional outcomes, the battle against poverty and malnutrition has to be fought on all fronts, to achieve a healthier and more equitable society.

## Author Contributions

All authors contributed to the study and the write-up.

## Conflict of Interest

The authors declare that the research was conducted in the absence of any commercial or financial relationships that could be construed as a potential conflict of interest.
